# Investigating the effect of tenuigenin on LPS-induced HPMEC dysfunction by inhibiting SRC activation based on network pharmacology and molecular docking

**DOI:** 10.1186/s41065-025-00574-6

**Published:** 2025-09-29

**Authors:** Jingchao Chen, Hao Pan, Jinchun Wang, Jing Han, Weihui Ma

**Affiliations:** 1 Emergency center, the Third Affiliated Clinical Hospital of Changchun University of Traditional Chinese Medicine, No.1643, Jingyue Street, Changchun City, 130000 Jilin China; 2 Department of pulmonology, the Third Affiliated Clinical Hospital of Changchun University of Traditional Chinese Medicine, Changchun City, 130000 Jilin China; 3 Department of Emergency, Changchun Hospital of Traditional Chinese Medicine, Changchun City, 130052 Jilin China

**Keywords:** Adult pneumonia, Tenuigenin, Steroid receptor coactivator, Molecular docking

## Abstract

**Background:**

Adult pneumonia is an infectious lung disease caused by bacteria, viruses, or other microorganisms and exhibits some degree of contagion. Tenuigenin, a bioactive compound derived from Polygala tenuifolia, possesses broad pharmacological effects, but its role in adult pneumonia remains incompletely understood.

**Methods:**

Bioinformatics and database analysis were employed to screen and analyze the Tenuigenin target genes relevant to adult pneumonia. Cell functions were assessed using cell counting kit-8 (CCK8), 5-ethynyl-2’-deoxyuridine (EdU) staining, transwell, tube formation, Fluo-4 calcium assay, and transepithelial electrical resistance (TER) assays. Protein levels were measured by western blot. Network pharmacology and molecular docking were employed to screen core target genes and verify binding interactions.

**Results:**

Tenuigenin targets in adult pneumonia were enriched in the pathways related to vascular permeability and calcium signaling. Tenuigenin mitigated lipopolysaccharide (LPS)-induced impairment of human pulmonary microvascular endothelial cell (HPMEC) viability, proliferation, migration, and angiogenesis, while attenuating LPS-induced increases in apoptosis, calcium ion, and reactive oxygen species (ROS) levels. Besides, Tenuigenin also attenuated the TER decrease and permeability increase caused by LPS exposure in HPMECs. Network pharmacology and molecular docking identified steroid receptor coactivator (SRC) as a core target of Tenuigenin, demonstrating binding to specific SRC amino acid residues. Tenuigenin also reduced LPS-induced increase in phosphor-SRC (p-SRC) expression. Crucially, after inhibition of SRC kinase activity, Tenuigenin no longer exerted significant protective effects against LPS-induced HPMEC injury and dysfunction.

**Conclusion:**

Tenuigenin alleviates LPS-induced injury and dysfunction of HPMECs by targeting the SRC pathway, providing a target for managing adult pneumonia.

**Supplementary Information:**

The online version contains supplementary material available at 10.1186/s41065-025-00574-6.

## Introduction

Adult pneumonia is a common respiratory infection characterized by inflammation of the lung parenchyma, typically caused by bacterial, viral, or fungal pathogens. It predominantly affects children and the elderly with chronic conditions [1]. Primary manifestations include cough, sputum production, fever, and pulmonary crepitus [2], imposing a significant socioeconomic burden [3]. Despite advancements in diagnosis, clinical management, treatment, and prevention, specific therapeutic strategies remain limited. Consequently, there is an urgent need to develop new methods to alleviate adult pneumonia.

Tenuigenin, the primary active constituent extracted from the roots of Polygala tenuifolia, exhibits diverse pharmacological activities, including anti-inflammatory, antioxidant, and neuroprotective effects [4, 5]. It has been used in treating neurological diseases [6], cerebral hemorrhage [7], and cancer [8]. Additionally, Tenuigenin exhibited anti-inflammatory activity in lipopolysaccharide (LPS)-stimulated macrophages by inhibiting mitogen-activated protein kinase (MAPK) and nuclear factor kappa-B (NF-κB) pathways while inducing the nuclear factor erythroid-derived-2-like 2/heme oxygenase-1 (Nrf2/HO-1) pathway [9]. In rats subjected to cecal ligation and puncture, Tenuigenin reduced oxidative stress and inflammation, thereby ameliorating acute lung injury [10]. Notably, Tenuigenin also showed protective effects against staphylococcus aureus-induced pneumonia [11], primarily confirming its efficacy against specific types of bacterial lung injury at the overall animal level. This study, for the first time, establishes a model of LPS-induced functional impairment in human pulmonary microvascular endothelial cells (HPMECs), directly investigating the effects of LPS on HPMECs and Tenuigenin’s intervention at the cellular level to elucidate underlying mechanisms more precisely.

Steroid receptor coactivator (SRC) is a tyrosine kinase and proto-oncogene up-regulated in multiple cancers, where it plays an oncogenic role [12]. The SRC-Zinc and ring finger 1 axis was shown to limit lung barrier damage by modulating the Toll like receptor 3 signaling [13]. Additionally, SRC was implicated in the resistance mediated by caffeic acid against pneumonia [14]. More importantly, network pharmacology analysis identified SRC as a core target of Tenuigenin in adult pneumonia. Nevertheless, its involvement in Tenuigenin’s therapeutic effect on adult pneumonia is unknown.

This study elucidates the mechanism by which Tenuigenin ameliorates adult pneumonia through the SRC pathway, utilizing a combination of network pharmacology, molecular docking, and molecular biology experiments. It aims to provide new insights for the clinical diagnosis and treatment of adult pneumonia.

## Materials and methods

### Target screening and bioinformatics analysis

Tenuigenin targets were obtained from the SwissTargetPrediction database (http://old.swisstargetprediction.ch/help.php), while adult pneumonia targets were predicted using the GeneCards database (https://www.genecards.org/). Intersection of these two target genes identified 78 common targets potentially involved in Tenuigenin’s action on adult pneumonia.

These 78 targets were input into the String website (https://cn.string-db.org/) to construct a protein-protein interaction (PPI) network, which was optimized using the Cytoscape software according to the Degree values.

The SangerBox (http://sangerbox.com/) was applied to conduct the Gene Ontology (GO) and Kyoto Encyclopedia of Genes and Genomes (KEGG) enrichment analyses of the 78 targets.

The TOP 10 hub genes were screened using the CytoHubba (a Cytoscape plugin) based on maximum neighborhood component (MNC), maximal clique centrality (MCC), and Degree values. SRC was identified as a top hub gene and selected for further investigation.

### Cell culture and treatment

The HPMECs supplied by Wuhan Pricella Biotechnology Co., Ltd. (Wuhan, China) were cultured in the HPMEC complete medium (Pricella) at 37℃ in a humidified 5% CO_2_ incubator. Cells were treated with Tenuigenin (0.5, 1, 2, 4, 8, and 16 µg/mL; MedChemExpress (MCE), Monmouth Junction, New Jersey, The United States of America (USA)), LPS (1 µg/mL, MCE), and/or SRC kinase inhibitor AZM475271 (20 µM, MCE) alone or together for 12 h as needed.

### Cell counting kit-8 (CCK8)

The HPMECs (5 × 10^3^ cells/well) were spread into 96-well plates and treated with different conditions. Following treatment with CCK8 solution (Beyotime, Shanghai, China), the HPMECs were incubated for 4 h. Then, the absorbance at 450 nm was measured using the EnVision Nexus Microplate Reader (Revvity, Shanghai, China).

### 5-Ethynyl-2’-deoxyuridine (EdU) staining

Cell proliferation was detected using the EdU Cell Proliferation Kit with Alexa Fluor 647 (Epizyme, Shanghai, China). After treatment, HPMECs in 48-well plates were incubated with 2×EdU working solution for 2 h. After that, the cells were treated with 4% paraformaldehyde for 15 min, permeabilized, treated with Click reaction solution for 30 min away from light, and nuclei were stained with 4’,6-diamidino-2’-phenylindole (DAPI) for 10 min avoiding light. The images were obtained under the microscope (Keyence, Shanghai, China).

### Transwell assay

The treated cells suspended in serum-free medium were spread into the upper chambers of the transwell plate, and the lower chambers were added with complete medium. After culturing for 24 h, the cells remaining on the upper chamber membrane surface were fixed with 4% paraformaldehyde for 30 min, and stained with 0.1% crystal violet for 10 min. Following air drying, the cells were photographed under the microscope (Keyence) and quantitatively studied using the Image J software.

### Tube formation assay

The starved HPMECs were re-suspended in HPMEC complete medium and inoculated into a 24-well plate pre-coated with Matrigel (Corning, Corning, New York (NY), USA). After the cells were treated with different conditions and cultured for 24 h, the images were obtained under the microscope (Keyence). Quantitative analysis was performed using the Angiogenesis Analyzer plugin in Image J software.

### Flow cytometry

For apoptosis, the cells treated with different conditions were collected in centrifuge tubes after centrifugation at 1000 g for 5 min, resuspended in Annexin V-fluorescein isothiocyanate (FITC) binding solution, and sequentially added with Annexin V-FITC and propidium iodide(PI). After reaction at 25℃ for 20 min away from light, the cells were detected using the flow cytometer (BeckmanCoulter, Pasadena, California (CA), USA). The steps were conducted according to the Annexin V-FITC Apoptosis Detection Kit (Beyotime).

For reactive oxygen species (ROS) levels, the collected cells were treated with 2’, 7’-dichlorodihydrofluorescein diacetate (DCFH-DA, Beyotime) at 37℃ for 20 min. Following washing with phosphate buffered saline (PBS), intracellular ROS levels were measured using the flow cytometer (BeckmanCoulter).

### Calcium ion level detection

The cells were spread into 24-well plates and treated as indicated. After washing with PBS, the cells were loaded with Fluo-4 staining solution and reacted at 37℃ for 30 min away from light. Fluorescence intensity, reflecting intracellular calcium concentration, was determined using the fluorescence microscope (Keyence). The above steps were conducted using the Fluo-4 Calcium Assay Kit (Beyotime).

### Transepithelial electrical resistance (TER) assay

The cells were seeded onto Matrigel (Corning)-coated transwell inserts and cultured to form confluent monolayers. After treatment, TER was tested under the Millipore Millicell ERS-2 Voltohmmeter (Millipore, Bedford, Massachusetts (MA), USA).

### Cell permeability assay

The treated HPMEC monolayers on transwell inserts were cultured for 24 h. Then, the medium in the upper chambers was replaced with the medium containing 10 µg/mL FITC-labeled Dextran (40 kDa, Beyotime), while the lower chambers contained PBS. After culturing for 1 h away from light, the PBS in the lower chambers was collected for the detection using a SpectraMax Gemini EM Fluorescence Microplate (Moleculardevices, Shanghai, China).

### Western blot

The total protein was isolated from the cells using the Proteintech Cell Total Protein Extraction Kit (Proteintech, Wuhan, China). Protein samples were mixed with sodium dodecyl sulfate-polyacrylamide gel electrophoresis (SDS-PAGE) protein Loading Buffer, denatured at 100℃ for 10 min, separated on SurePAGE™ Protein Precast Gels (Genscript, Nanjing, China) at 140 V for 1 h, and transferred onto polyvinylidene fluoride (PVDF) membranes (Beyotime). Membranes were subsequently closed by the 5% skim milk and incubated with anti-vascular endothelial cadherin (VE-cadherin) (1:1000, #2500, Cell signaling technology, Danvers, MA, USA), anti-Zona occludens 1 (ZO-1) (1:1000, #13663, Cell signaling technology), anti-phosphor-SRC (p-SRC) (1:5000, ab185617, Abcam, Cambridge, United Kingdom (UK)), anti-SRC (1:2000, 25978-1-AP, Proteintech), or anti-glyceraldehyde-3-phosphate dehydrogenase (GAPDH) (1:2500, ab9485, Abcam) at 4℃ overnight. Membranes were then incubated with HRP-conjugated Goat Anti-Rabbit IgG (H + L) (1:10000, SA00001-2, Proteintech) at 37℃ for 2 h. Protein bands were visualized using the ChemiDoc™ Imaging System (Bio-rad, Hercules, CA, USA), and band intensities were quantified using the Image Lab software.

### Molecular Docking

The three dimensions (3D) structures of Tenuigenin and SRC were downloaded from the Pubchem database (https://pubchem.ncbi.nlm.nih.gov/) and Protein Data Bank (PDB) database (https://www.rcsb.org/), respectively. Structures were prepared for docking using the AutodockTools, including dehydration, hydrogenation, amino acid optimization, and charge calculation and exported as pdbqt files. The Autodock Vina software was applied to conduct molecular docking and obtain the binding energy. The PyMOL software and Discovery Studio 4.5 software were used to visualize the interaction diagrams, respectively.

### Statistical analysis

Data were presented as mean ± standard deviation (SD). The GraphPad Prism 8 (GraphPad Software, San Diego, CA, USA) was applied to conduct data analysis. Differences between groups were assessed by one-way analysis of variance (ANOVA). *P* < 0.05 was deemed statistically significant.

## Results

### Screening the targets of tenuigenin in adult pneumonia

**Fig. 1:**
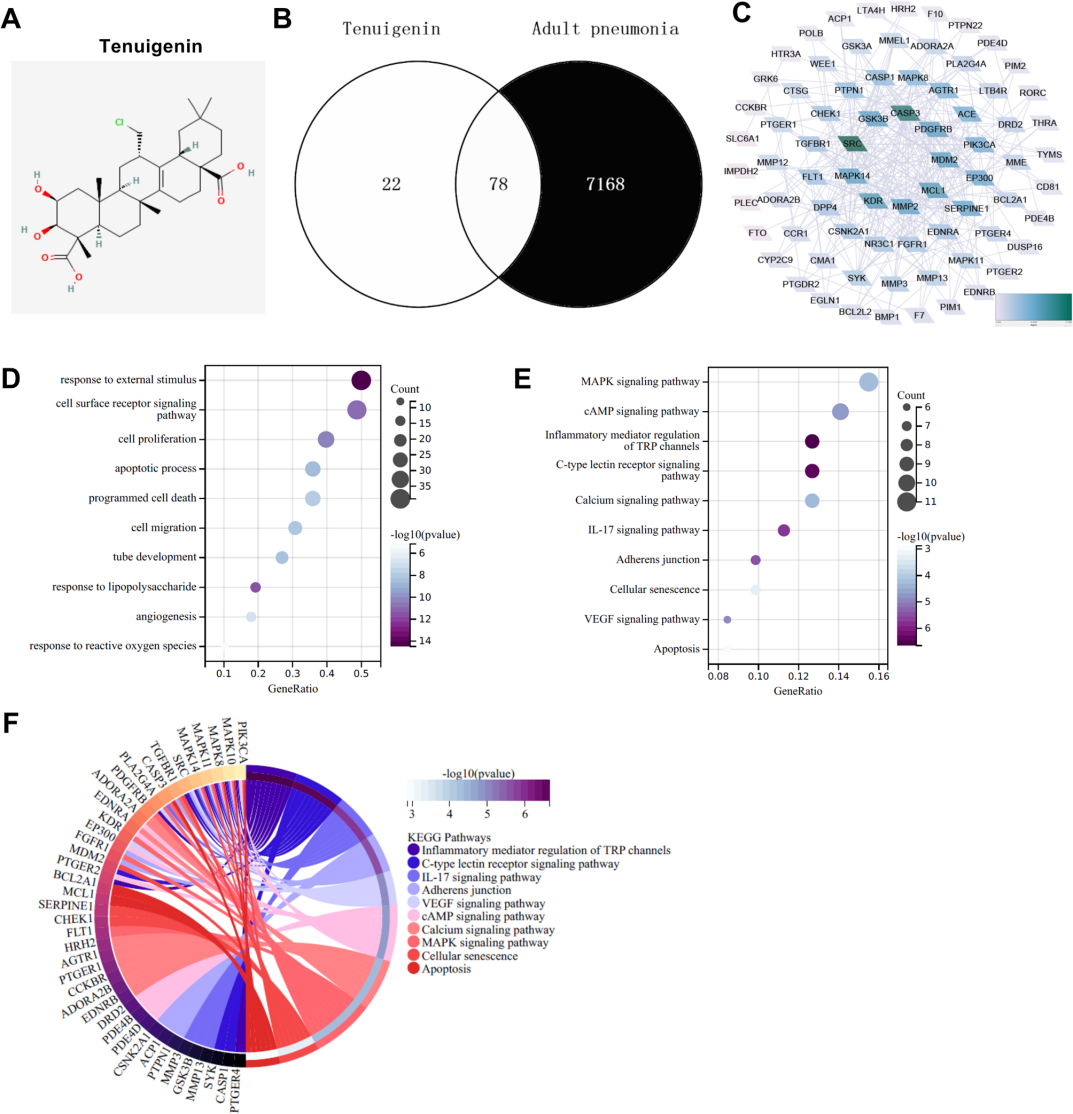
The target screen of Tenuigenin in adult pneumonia. (**A**) The image showed the 2D structure of Tenuigenin. (**B**) The Venn diagram showed that 78 targets were screened from the intersection of the 100 Tenuigenin targets from the SwissTargetPrediction database and the 7246 adult pneumonia targets from the GeneCards database. (**C**) The PPI network of the 78 common targets was analyzed by the String website and optimized by the Cytoscape software (The darker the color, the higher the Degree value). (**D**) The bubble diagram showed the GO enrichment analysis of the 78 common targets conducted by the SangerBox. (**E**) The bubble diagram showed the KEGG enrichment analysis of the 78 common targets conducted by the SangerBox. (**F**) The loop graph showed the KEGG enrichment analysis of the 78 common targets conducted by the SangerBox

The 2D structure diagram of Tenuigenin is shown in Fig. 1A. Intersection analysis of 100 Tenuigenin targets from the SwissTargetPrediction database and 7246 adult pneumonia targets from the GeneCards database yielded 78 common targets (Fig. 1B). The PPI network of these 78 targets is depicted in Fig. 1C. GO enrichment analysis displayed enrichment in biological processes including response to external stimulus, cell surface receptor signaling pathway, cell proliferation, apoptotic process, programmed cell death, cell migration, tube development, response to lipopolysaccharide, angiogenesis, and response to reactive oxygen species (Fig. 1D). KEGG enrichment analysis revealed significant enrichment in the pathways such as Calcium signaling pathway, Adherens junction, Cellular senescence, VEGF signaling pathway, and Apoptosis (Fig. 1E-F). These pathways are implicated in endothelial cell (EC) damage associated with adult pneumonia and lung injury.

### Tenuigenin enhances the viability of LPS-induced HPMECs

**Fig. 2:**
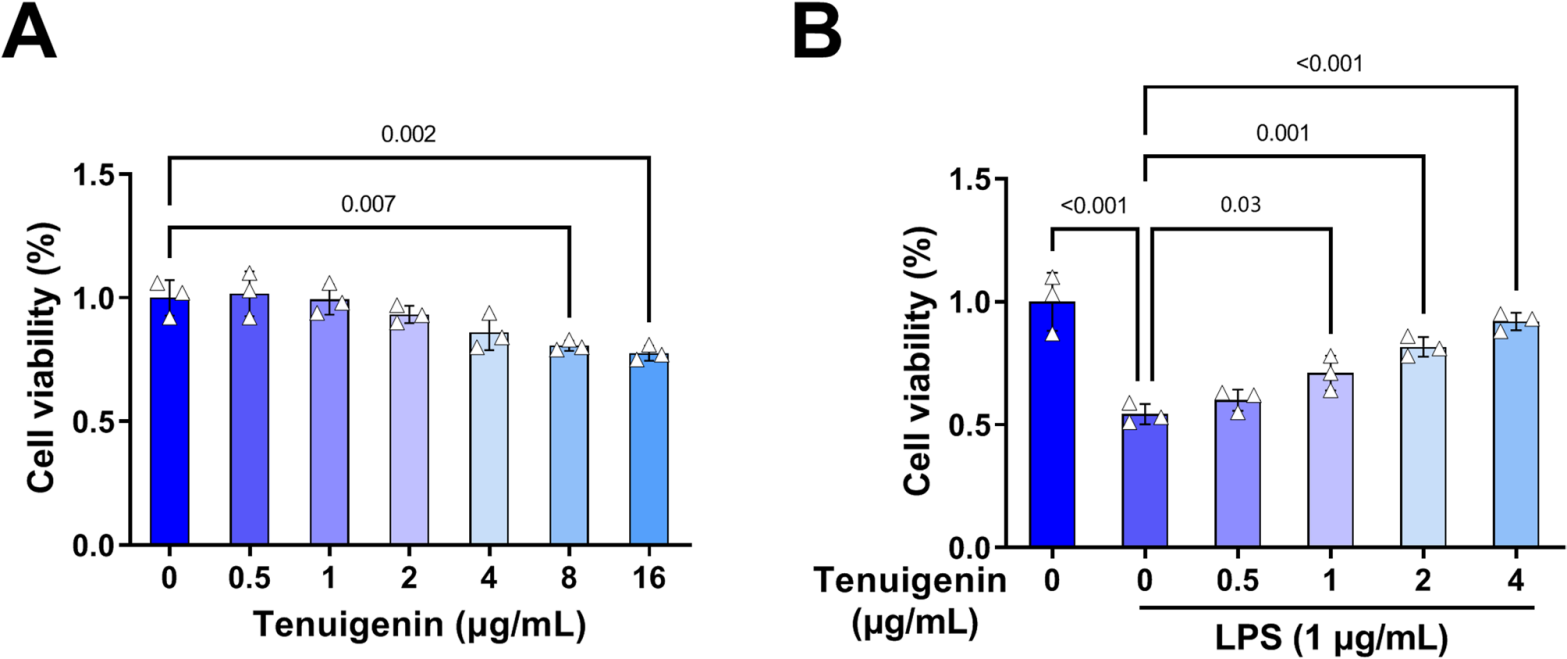
Tenuigenin promotes the viability of LPS-induced HPMECs. (**A**) CCK8 was used to detect the viability of HPMECs treated with Tenuigenin at 0, 0.5, 1, 2, 4, 8, and 16 µg/mL. (**B**) CCK8 was used to detect the viability of HPMECs treated with Tenuigenin (0, 0.5, 1, 2, and 4 µg/mL) and LPS (1 µg/mL). All experiments were repeated three times. ^*^P < 0.05, ^**^P < 0.01, ^***^P < 0.001

Treatment of HPMECs with increasing concentrations of Tenuigenin (0, 0.5, 1, 2, 4, 8, and 16 µg/mL) showed no effect on viability at 1, 2, and 4 µg/mL, but significantly reduced viability at 8 and 16 µg/mL (Fig. 2A). Meanwhile, co-treatment with different concentrations of Tenuigenin (0, 0.5, 1, 2, and 4 µg/mL) and LPS (1 µg/mL) demonstrated that Tenuigenin attenuated the LPS-induced decline in HPMEC viability (Fig. 2B). Therefore, these indicates Tenuigenin’s protective effect.

### Tenuigenin reverses LPS-induced apoptosis, calcium ions, and ROS levels

**Fig. 3:**
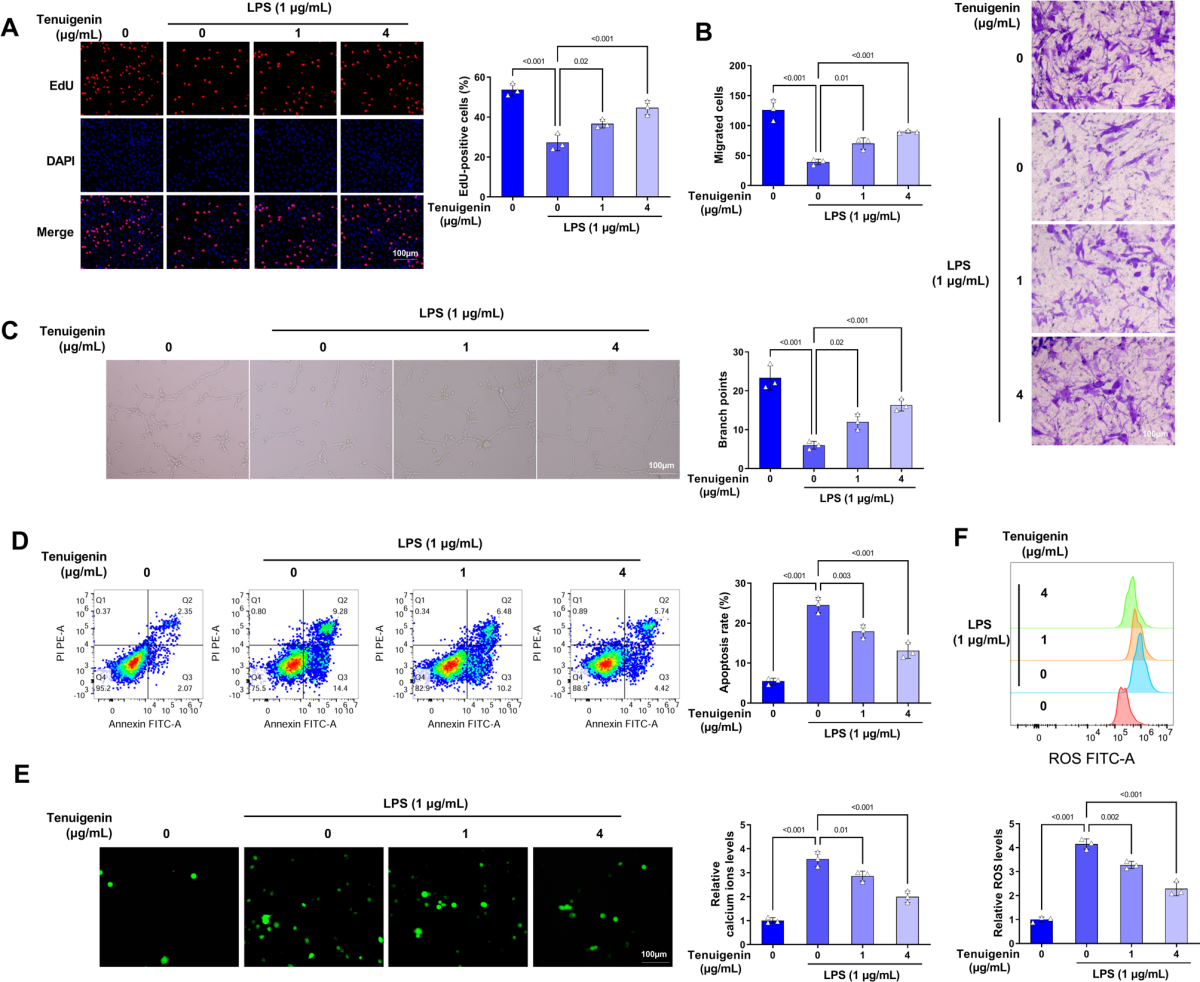
Tenuigenin mitigates LPS-induced HPMEC dysfunction. The HPMECs were co-treated with Tenuigenin (0, 1, and 4 µg/mL) and LPS (1 µg/mL). (**A**) EdU staining was applied to measure the EdU-positive cells. (**B**) The migrated cells were determined by transwell assay. (**C**) The angiogenesis was examined by tube formation assay. (**D**) Flow cytometry was employed to detect apoptosis. (**E**) The calcium ions levels were evaluated by the Fluo-4 Calcium Assay Kit. (**F**) The ROS levels were estimated by flow cytometry. All experiments were repeated three times. ^*^P < 0.05, ^**^P < 0.01, ^***^P < 0.001

To examine the effects of Tenuigenin on LPS-induced HPMEC functions, the experiments were performed. Tenuigenin mitigated LPS-induced reductions in EdU-positive cells, migrated cells, and angiogenesis (Fig. 3A-C). Besides, Tenuigenin suppressed LPS-mediated HPMEC apoptosis (Fig. 3D). Meanwhile, the increased intracellular calcium ions and ROS levels caused by LPS were also decreased after Tenuigenin treatment (Fig. 3E-F). Collectively, Tenuigenin alleviates the LPS-caused HPMEC injury.

### Tenuigenin preserves barrier function in LPS-induced HPMECs

**Fig. 4:**
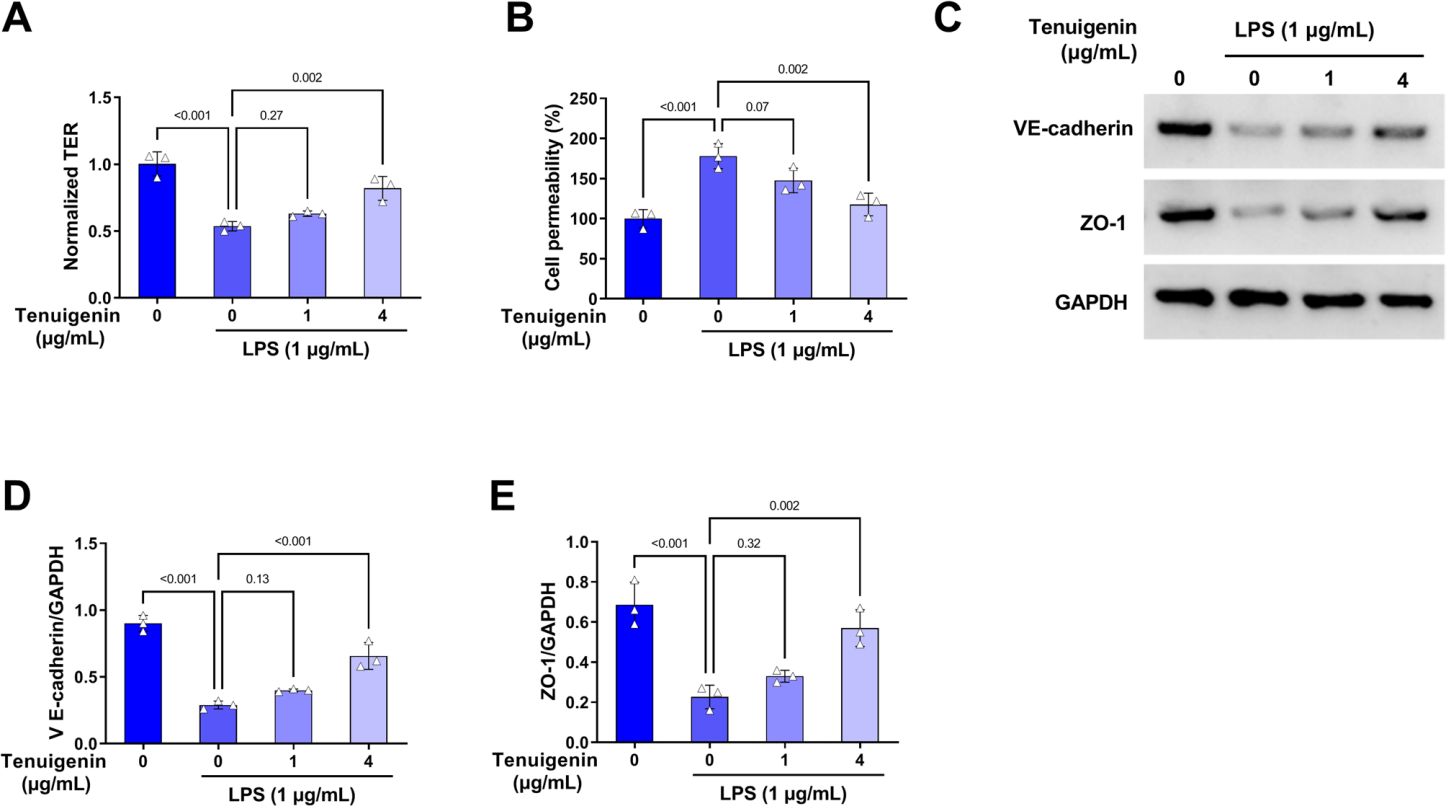
Tenuigenin impairs the cell permeability of LPS-induced HPMECs. The HPMECs were co-treated with Tenuigenin (0, 1, and 4 µg/mL) and LPS (1 µg/mL). (**A**) The TER was measured by the TER method. (**B**) The cell permeability was determined by transwell assay. (**C-E**) Western blot was applied to examine the protein expression of VE-cadherin and ZO-1. All experiments were repeated three times. ^*^P < 0.05, ^**^P < 0.01, ^***^P < 0.001

The depressed TER and elevated permeability of HPMECs induced by LPS were alleviated with the treatment of Tenuigenin (Fig. 4A-B). Consistent with this, western blot revealed that LPS down-regulated the expression of key barrier proteins VE-cadherin and ZO-1, effect that was reversed by Tenuigenin exposure (Fig. 4C-E). Consequently, Tenuigenin protects against LPS-induced HPMEC barrier dysfunction.

### Molecular Docking of tenuigenin and SRC

**Fig. 5:**
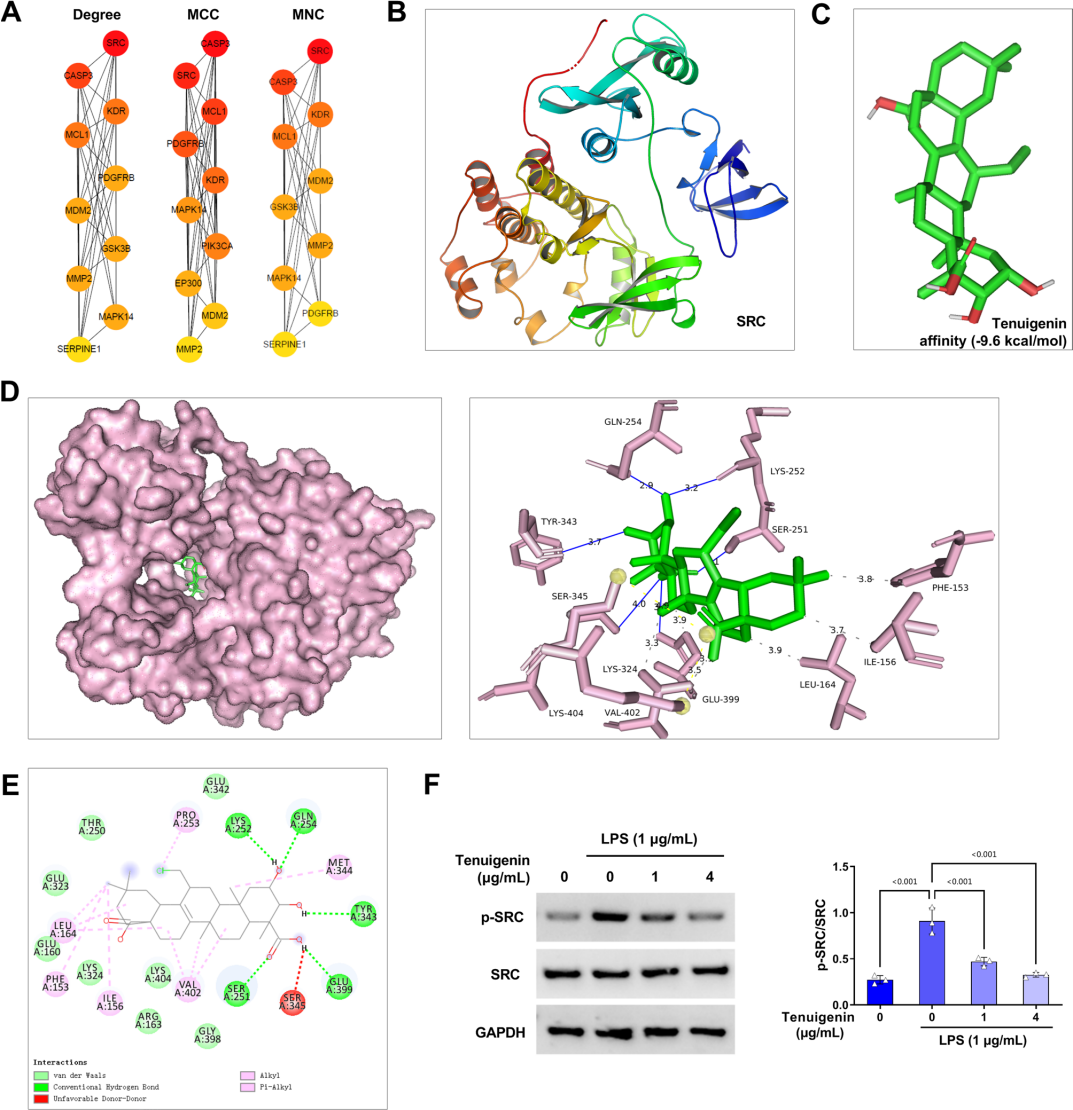
SRC is the target of Tenuigenin in adult pneumonia. (**A**) The TOP 10 hub genes of Tenuigenin in adult pneumonia were screened according to the MNC, MCC, and Degree values of the CytoHubba. (**B**) The image showed the 3D structure of SRC. (**C**) The 3D image showed the optimal conformation of Tenuigenin and SRC (affinity: -9.6 kcal/mol) visualized by the PyMOL software. (**D**) The 3D combination image of Tenuigenin and SRC was visualized by the PyMOL software. (**E**) The 2D combination image of Tenuigenin and SRC was visualized by the Discovery Studio 4.5 software. (**F**) Western blot was applied to examine the protein expression of SRC and p-SRC in HPMECs co-treated with Tenuigenin (0, 1, and 4 µg/mL) and LPS (1 µg/mL). All experiments were repeated three times. ^***^P < 0.001

To explore the mechanism of Tenuigenin on adult pneumonia, the targets of Tenuigenin was further screened. Analysis of the TOP 10 hub genes from the PPI network using the MNC, MCC, and Degree values of CytoHubba identified SRC as the TOP gene by MNC and Degree criteria (Fig. 5A). The 3D structure diagram of SRC is shown in Fig. 5B. Meanwhile, molecular docking revealed a favorable binding conformation between Tenuigenin and SRC, with a binding energy of -9.6 kcal/mol (Fig. 5C). Visualization of the 3D and 2D interaction diagrams is presented in Fig. 5D-E. Importantly, Tenuigenin treatment reversed the LPS-induced increase in p-SRC expression (Fig. 5F).

### Tenuigenin mitigates HPMEC injury and dysfunction caused by LPS via the SRC pathway

**Fig. 6:**
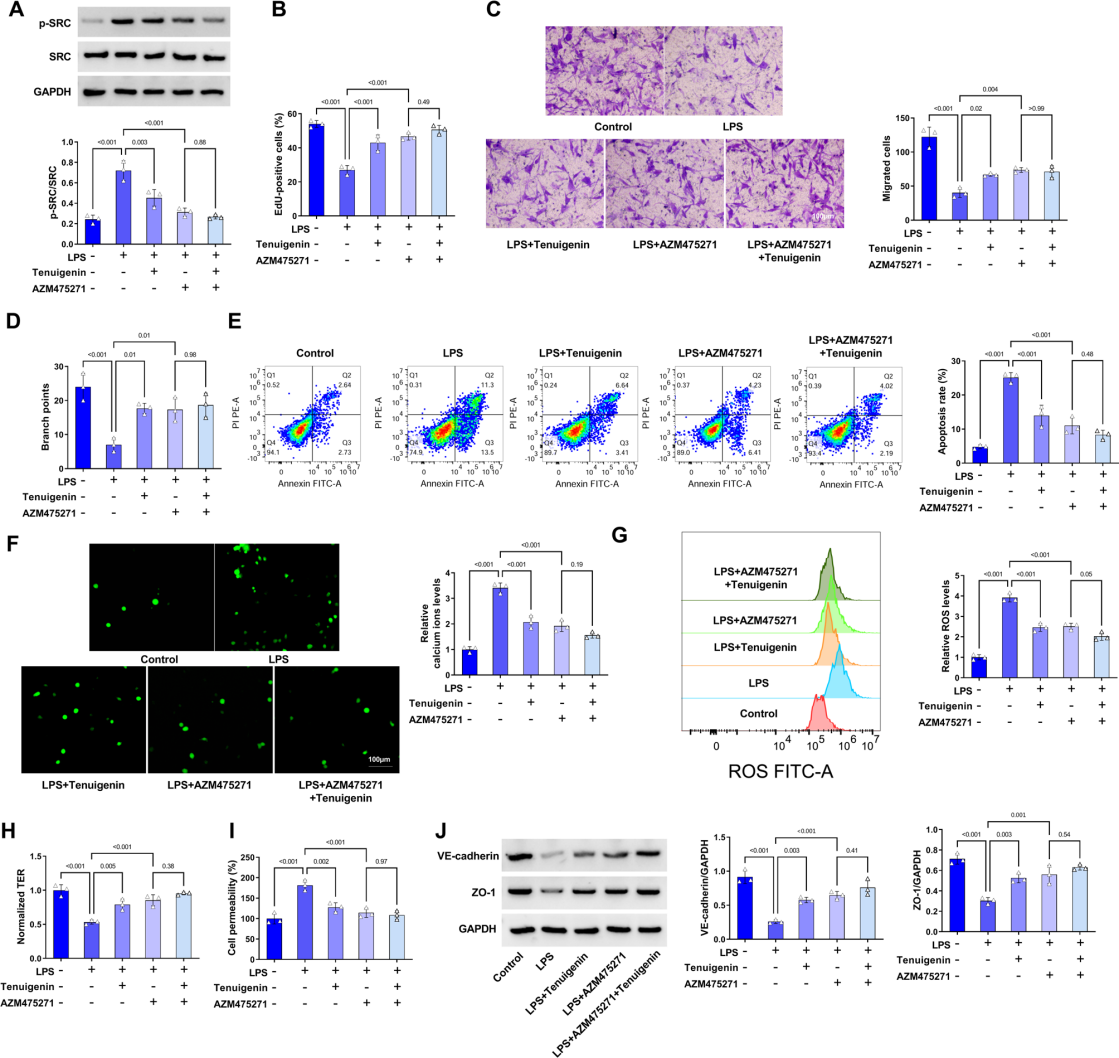
Tenuigenin weakens LPS-induced HPMEC injury and dysfunction through the SRC pathway. The HPMECs were treated with LPS, Tenuigenin, and AZM475271. (**A**) Western blot was used to examine the protein expression of SRC and p-SRC. (**B**) EdU staining was applied to measure the EdU-positive cells. (**C**) The migrated cells were determined by transwell assay. (**D**) The angiogenesis was examined by tube formation assay. (**E**) Flow cytometry was employed to detect apoptosis. (**F**) The calcium ions levels were evaluated by the Fluo-4 Calcium Assay Kit. (**G**) The ROS levels were estimated by flow cytometry. (**H**) The TER was measured by the TER method. (**I**) The cell permeability was determined by transwell assay. (**J**) Western blot was employed to examine the protein expression of VE-cadherin and ZO-1. All experiments were repeated three times. ^*^P < 0.05, ^**^P < 0.01, ^***^P < 0.001

To better explain the functional role of Tenuigenin and SRC in LPS-induced HPMEC injury, the SRC kinase inhibitor AZM475271 was used for mechanistic validation experiments. Both Tenuigenin and AZM475271 attenuated the LPS-induced increase in p-SRC expression; their combination showed a trend towards further reduction, but this was not statistically significant (Fig. 6A). The protective effects of Tenuigenin against LPS-induced impairment of proliferation, migration, and angiogenic ability were mimicked by AZM475271. Notably, the combination of Tenuigenin and AZM475271 did not yield significantly greater protection than Tenuigenin alone (Fig. 6B-D). Similarly, both Tenuigenin and AZM475271 significantly attenuated LPS-induced apoptosis in HPMECs, along with reducing intracellular calcium overload and ROS overproduction. However, the combination treatment did not significantly enhance these protective effects compared to Tenuigenin alone (Fig. 6E-G). Meanwhile, exposure to Tenuigenin and AZM475271 alone or in combination promoted TER and inhibited permeability of HPMECs, while the effects of Tenuigenin on these functions were not significantly different after inhibition of SRC kinase activation (Fig. 6H-J). Beyond that, the inhibitory effects of Tenuigenin on LPS-induced HPMEC injury and dysfunction were partially reversed after SRC overexpression (Supplementary Fig. 1). Taken together, Tenuigenin attenuates LPS-induced HPMEC injury and dysfunction primarily by targeting the SRC pathway.

## Discussion

Compared with Western medicine treatment, traditional Chinese medicine (TCM) treatment is often favored for its lower side effects and costs. TCM offers significant benefits in preventing and treating pneumonia through multi-targets and multi-pathway regulation [15]. Tenuigenin exhibits a wide range of pharmacological activities. Studies had shown it alleviateds amyloid-β peptide (Aβ_1−42_)-induced neuronal cell damage in vitro [16] and improved motor function recovery in rats with spinal cord injury by inhibiting autophagy in vivo [17]. Notably, Tenuigenin ameliorated LPS-induced acute lung injury, primarily by inhibiting the production of inflammatory factors [18]. Tenuigenin also inhibited epithelial cell oxidation and senescence through the Sirtuin 1/peroxisome proliferators-activated receptor γ (PPAR-γ) coactivator 1 alpha pathway, alleviating pulmonary fibrosis [19], and protected against Staphylococcus aureus-induced pneumonia in mice [11]. In this study, it was found that Tenuigenin targets in adult pneumonia were mainly enriched in pathways related to vascular leakage, inflammation, and calcium signaling.

Calcium ions are crucial intracellular second messengers. In ECs, abnormal elevation of calcium ions (calcium overload) leads to dysfunction and damage [20, 21]. Specifically, calcium overload activates proteases (e.g., calpain) and phospholipase, causing membrane destruction, cytoskeleton disintegration, and apoptosis [20, 21]. Beyond that, it compromises EC barrier function, increasing vascular permeability and exacerbating inflammation and tissue damage [22, 23]. More importantly, the core pathology of adult pneumonia involves inflammatory exudation and pulmonary edema resulting from disruption of the pulmonary microvascular endothelial barrier. HPMECs, central to the blood-air barrier, were the focus of this study. Their dysfunction (such as increased permeability and calcium signal disruption) directly facilitates vascular fluid and inflammatory cell extravasation, triggering alveolar edema and inflammatory cascades [22, 24, 25]. LPS-induced decrease in HPMEC viability, increased apoptosis, and reduced barrier protein (VE-cadherin/ZO-1) expression, calcium overload, and ROS elevation mimic the pathological state of endothelial damage in adult pneumonia. Calcium overload and ROS activate pro-inflammatory pathways such as NF-κB, further aggravating lung tissue damage [20–23]. Therefore, targeting HPMEC dysfunction is a critical for intervening in pneumonia-induced lung damage. In this study, decreased viability and increased apoptosis was observed in HPMECs, which would disrupt endothelial integrity, facilitating pathogen and inflammatory factors penetration into lung tissue, thereby directly exacerbating pulmonary parenchymal infection [26]. LPS-induced TER decrease and FITC-glucan leakage directly reflect endothelial barrier disintegration, mirroring the alveolar-capillary leakage syndrome in clinical pneumonia patients and underpinning pulmonary edema [24]. Besides, calcium overload activates damaging proteases and induces mitochondrial ROS bursts, exacerbating cell death and barrier failure through oxidative stress [20, 21]. Overall, LPS induced the decrease of HPMEC viability, proliferation, migration, and angiogenesis, as well as the increase of apoptosis, calcium overload, and ROS, indicating HPMEC dysfunction. Tenuigenin effectively reversed these effects, particularly mitigating LPS-induced hyperpermeability, calcium overload, and oxidative stress. This action essentially repairs the microvascular barrier collapse and dampens inflammatory amplification central to adult pneumonia pathology, aligning with clinical treatment goals (reducing pulmonary edema and controlling inflammation) and providing experimental support for therapeutic strategies targeting endothelial dysfunction.

In recent years, network pharmacology has become a valuable tool in TCM research, providing multi-directional insights for the diagnosis and treatment of diseases [27]. Molecular docking, based on structural biology, is essential for predicting interactions between small molecules and target proteins [28]. Using these approaches, a strong interaction between Tenuigenin and SRC was identified.

SRC kinase plays an important role in EC injury. Under pathological conditions such as inflammation and ischemia-reperfusion, SRC activation promotes the inflammation, oxidative stress, and apoptosis of ECs [29, 30]. SRC kinase also critically regulates the barrier function and vascular permeability of ECs, contributing to tissue injury and inflammation [31–34]. Consequently, SRC is closely linked to lung injury, aggravating it by regulating inflammation, disrupting barrier integrity, and influencing apoptosis and proliferation balance. It was hypothesized that Tenuigenin protected HPMECs by targeting SRC, which was functionally verified using the SRC kinase inhibitor AZM475271.

In conclusion, this study proposes a novel mechanism by which Tenuigenin alleviates adult pneumonia. These findings indicate that Tenuigenin inhibits LPS-induced injury and dysfunction of HPMECs by inhibiting SRC pathway activity, revealing that the SRC pathway serves as a potential new target for intervening in LPS-induced pulmonary endothelial injury for the first time, laying a crucial theoretical foundation for developing more precise treatment strategies for adult pneumonia based on Tenuigenin or its derivatives. While in vitro cell models, particularly using a single cell type (such as HPMECs in this study), are invaluable for elucidating specific cellular mechanisms, they cannot fully mimic the complex in vivo microenvironment or simulate the interactions between multiple cell types (such as immune cells, epithelial cells, and fibroblasts) during adult pneumonia progression. The lack of in vivo experimental data (such as LPS-induced mouse pneumonia model) in the current research is a significant limitation, hindering direct extrapolation of in vitro findings to the whole disease process. In future studies, animal models will be used to verify the core findings presented here and explore Tenuigenin’s effects within the physiologically complex environment of adult pneumonia, providing a more comprehensive assessment of its therapeutic potential.

## Supplementary Information

Below is the link to the electronic supplementary material.

**Supplementary figure 1**: SRC overexpression reverses the effects of tenuigenin on LPS-induced HPMEC injury and dysfunction. The HPMECs were treated with LPS, Tenuigenin, and oe-SRC. (**A**) EdU staining was applied to measure the EdU-positive cells. (**B**) The migrated cells were determined by transwell assay. (**C**) The angiogenesis was examined by tube formation assay. (**D**) Flow cytometry was employed to detect apoptosis. (**E**) The calcium ions levels were evaluated by the Fluo-4 Calcium Assay Kit. (**F**) The ROS levels were estimated by flow cytometry. (**G**) The TER was measured by the TER method. (**H**) The cell permeability was determined by transwell assay. All experiments were repeated three times. ^*^P < 0.05, ^**^P < 0.01, ^***^P < 0.001

Supplementary Material 2

## Data Availability

No datasets were generated or analysed during the current study.
