# Supplementary figures and images for: Investigating the effect of tenuigenin on LPS-induced HPMEC dysfunction by inhibiting SRC activation based on network pharmacology and molecular docking
Source: Hereditas. 2025 Sep 29;162:196. doi: 10.1186/s41065-025-00574-6 (PMC12481841; doi:10.1186/s41065-025-00574-6)

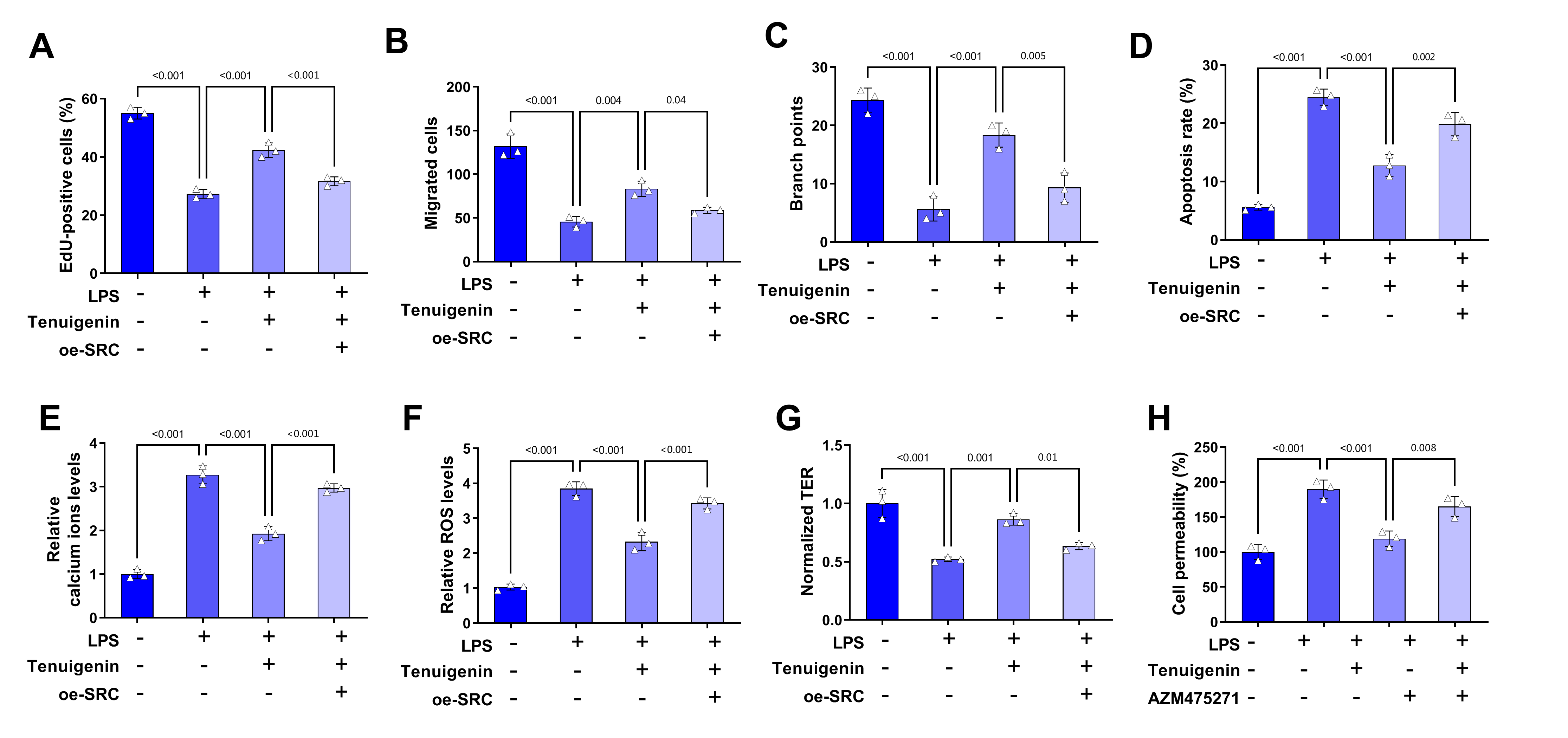

Supplement: Supplementary file 1 — Supplementary figure 1: SRC overexpression reverses the effects of tenuigenin on LPS-induced HPMEC injury and dysfunction. The HPMECs were treated with LPS, Tenuigenin, and oe-SRC. (A) EdU staining was applied to measure the EdU-positive cells. (B) The migrated cells were determined by transwell assay. (C) The angiogenesis was examined by tube formation assay. (D) Flow cytometry was employed to detect apoptosis. (E) The calcium ions levels were evaluated by the Fluo-4 Calcium Assay Kit. (F) The ROS levels were estimated by flow cytometry. (G) The TER was measured by the TER method. (H) The cell permeability was determined by transwell assay. All experiments were repeated three times. *P < 0.05, **P < 0.01, ***P < 0.001 [file 41065_2025_574_MOESM1_ESM.tif]

**Fig 4C**

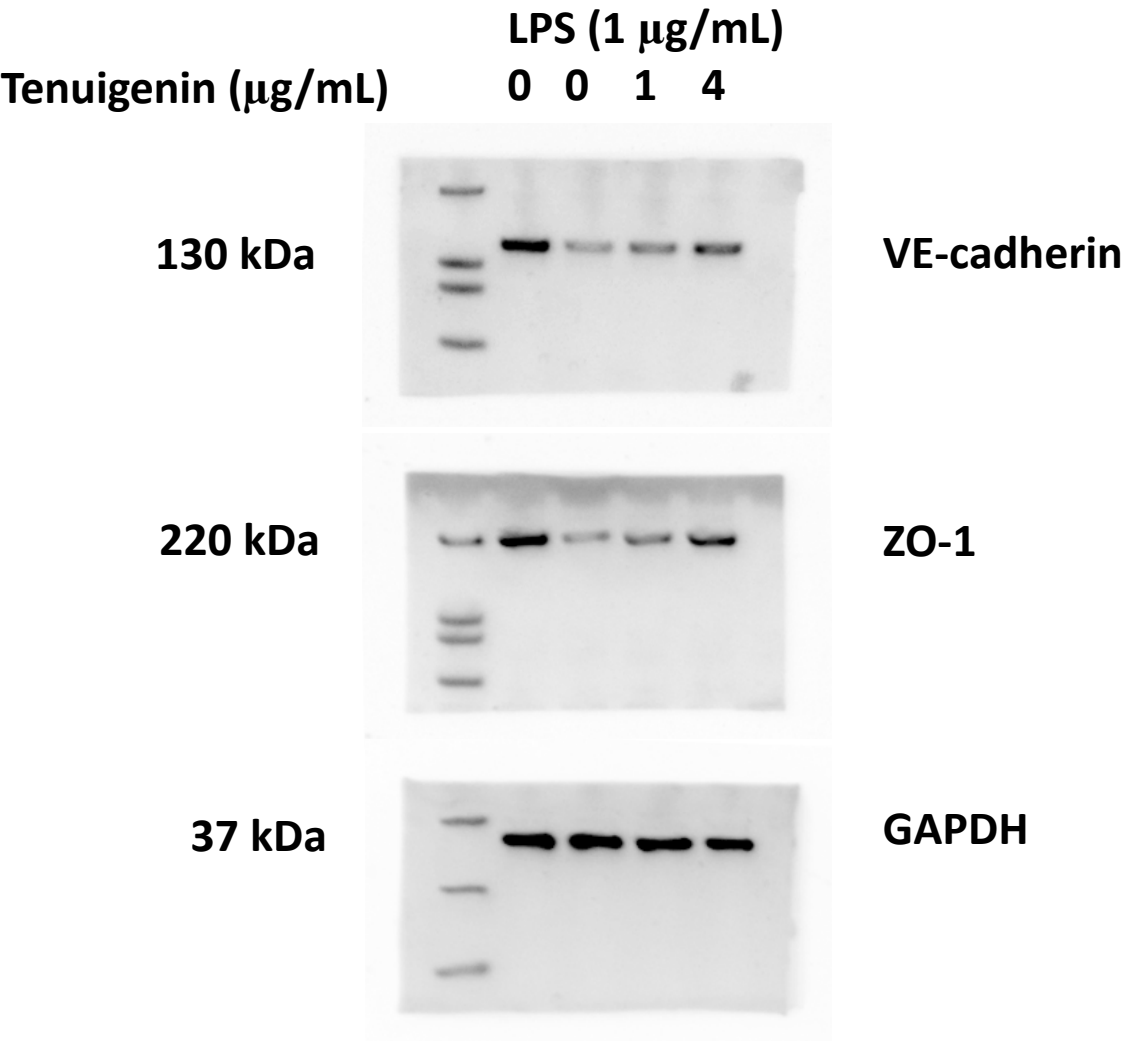

**Fig 5F**

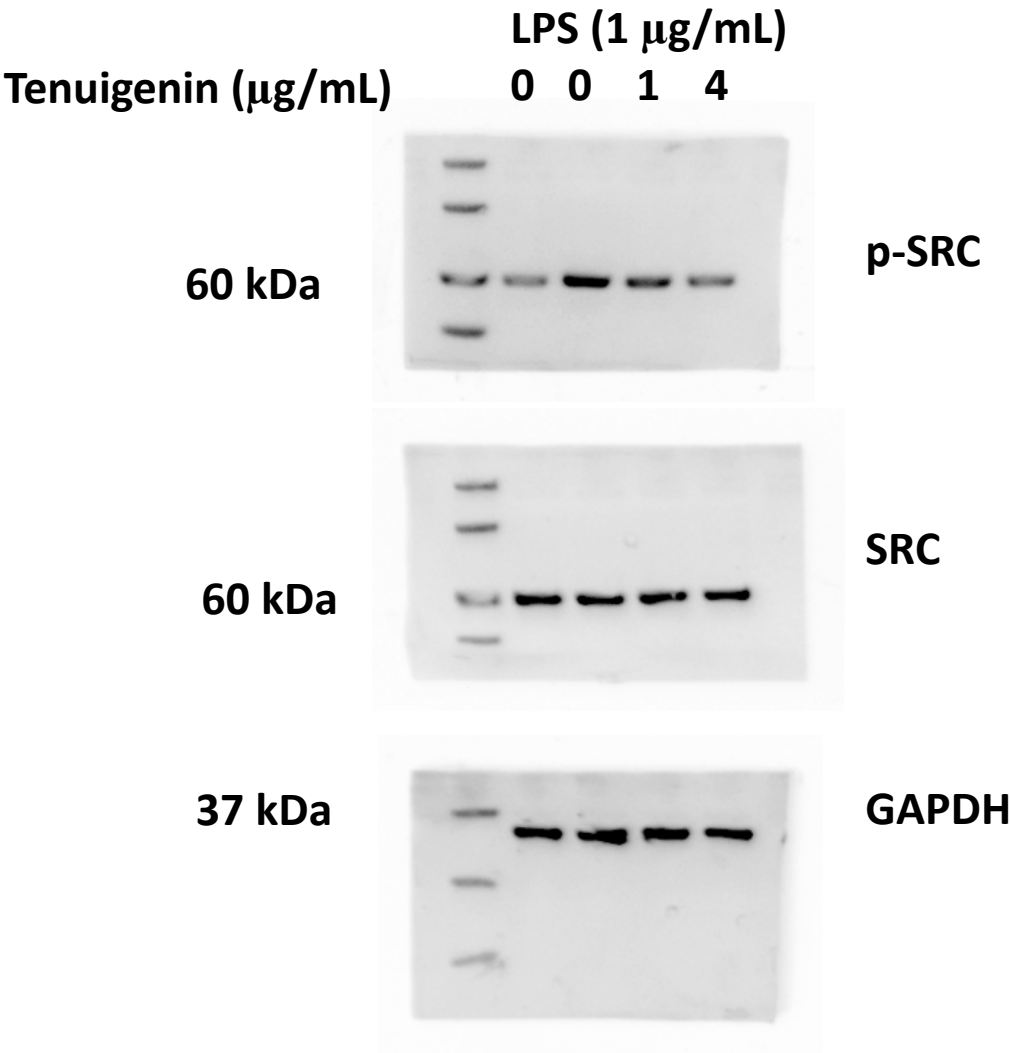

Fig 6A

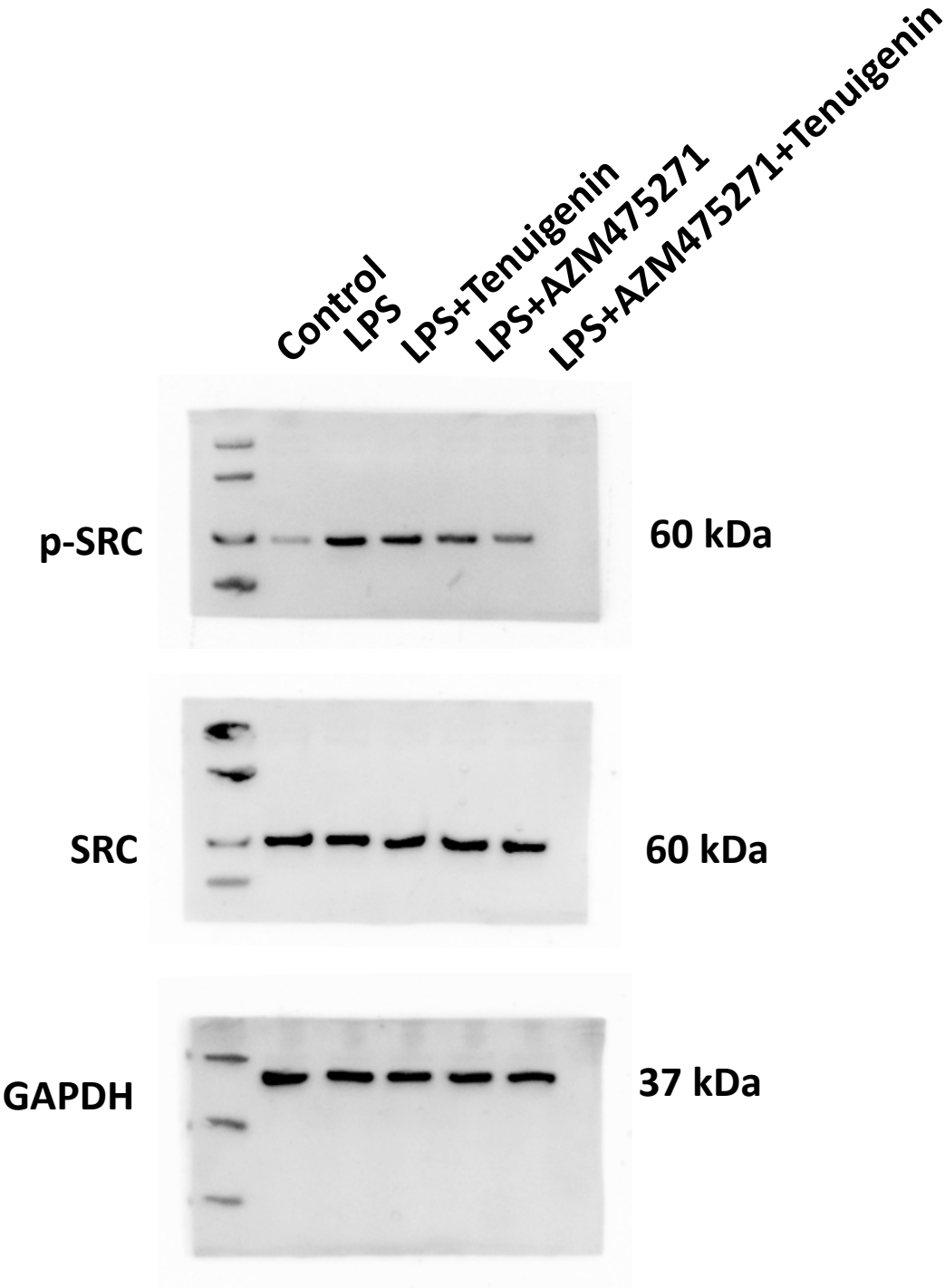

Fig 6J

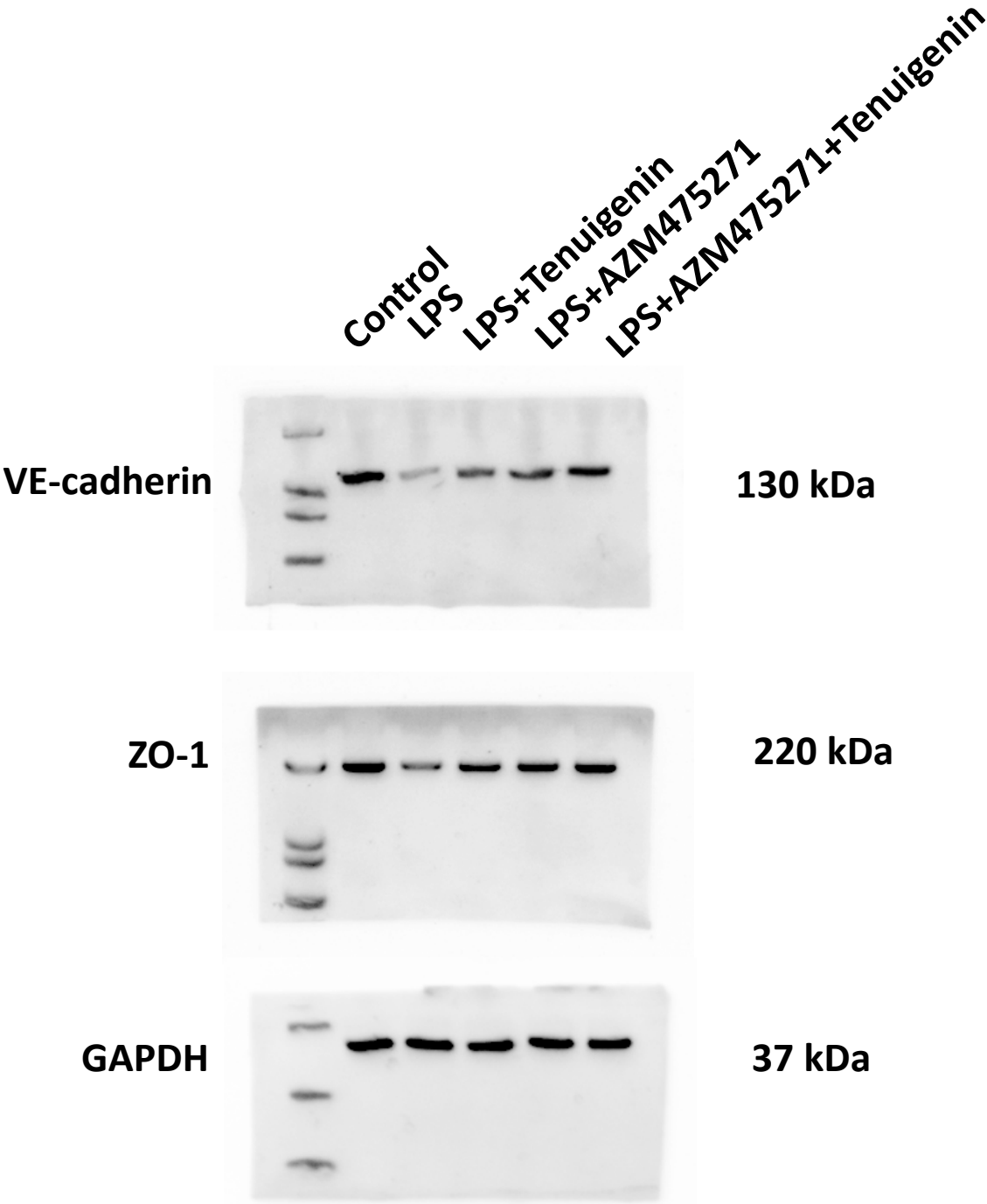

Supplement: Supplementary file 2 — Supplementary Material 2 [file 41065_2025_574_MOESM2_ESM.pdf]
